# STX2 promotes colorectal cancer metastasis through a positive feedback loop that activates the NF-κB pathway

**DOI:** 10.1038/s41419-018-0675-x

**Published:** 2018-05-31

**Authors:** Yongxia Wang, Honghai Xu, Hongli Jiao, Shuyang Wang, Zhiyuan Xiao, Yali Zhao, Jiaxin Bi, Wenting Wei, Shanshan Liu, Junfeng Qiu, Tingting Li, Li Liang, Yaping Ye, Wenting Liao, Yanqing Ding

**Affiliations:** 1 Department of Pathology, Nanfang Hospital, Southern Medical University, Guangzhou, 510515 Guangdong China; 2 Department of Pathology, School of Basic Medical Sciences, Southern Medical University, Guangzhou, Guangdong China; 3 Guangdong Provincial Key Laboratory of Molecular Tumor Pathology, Guangzhou, Guangdong China; 4 Department of Pathology, School of Basic Medical Sciences, Xinxiang Medical University, Xinxiang, Henan China

## Abstract

Metastatic progression is the main contributor to the poor prognosis of colorectal cancer (CRC). Thus, identifying the determinants of CRC metastasis will be of great significance. Based on our previous bioinformatics analysis, Syntaxin2 (STX2) may be upregulated and correlated with the poor prognosis of CRC patients. In this study, we found that STX2 expression was associated with CRC invasion and metastasis and poor patient survival. Gain- and loss-of-function analyses demonstrated that STX2 functioned as a key oncogene by promoting CRC invasion and metastasis. Mechanistically, STX2 selectively interacted with tumor necrosis factor receptor-associated factor 6 (TRAF6) and activated the nuclear transcription factor-κB (NF-κB) signaling pathway. Furthermore, chromatin immunoprecipitation (ChIP) analysis revealed that NF-κB directly bound to the STX2 promoter and drove STX2 transcription. Therefore, STX2 activated the NF-κB pathway, and in turn, NF-κB increased STX2 expression, forming a positive signaling loop that eventually promoted CRC metastasis. Collectively, our results reveal STX2 as a crucial modulator of the aggressive CRC phenotype and highlight STX2 as a potential prognostic biomarker and therapeutic target for combating CRC metastasis.

## Introduction

Colorectal cancer (CRC) is the third most prevalent cancer and the main cause of cancer-related death worldwide^1^. The poor prognosis of patients with CRC is largely due to the metastatic progression of CRC^2^. To date, efforts aimed at increasing cure rates after surgery have been focused on combined chemotherapy administration as a means of preventing metastasis. Such therapy reduces metastatic relapse by ~7%^3^. The high prevalence and lack of effective adjuvant therapeutics for this disease demand a greater understanding of the biology of CRC progression.

Metastatic progression is a complex multistep process involving alterations in the dissemination, invasion, survival, and growth of new cancer cell colonies, which are regulated by a complicated network of intra- and inter-cellular signal transduction cascades^4,5^. Although, alterations to multiple genes and signaling pathways, such as the mutational inactivation of the adenomatous polyposis coli (APC) gene, activation of the Kirsten rat sarcoma viral oncogene (KRAS), and activation of the Wnt or NF-κB signaling pathway, are responsible for the progression of CRC, metastasis remains the most poorly understood component of cancer pathogenesis^6^. Therefore, efforts to elucidate the mechanisms of CRC metastasis will enable the development of effective approaches to reduce CRC-associated mortality.

Syntaxin2 (STX2) is an important member of the syntaxin family and is highly conserved^7^. STX2 anchors onto the cytomembrane via its C-terminal domain and functions via its N-terminal domain^8^. STX2 participates in the tumorigenesis or metastasis of several cancers by regulating the expression of several key oncogenes, such as β-catenin and MMP9^9–11^. However, the biological functions of STX2 and the molecular mechanisms underlying these functions in CRC progression remain unknown.

NF-κB signaling pathway hyperactivation plays critical roles in different malignant progression-associated processes, including tumorigenesis, angiogenesis, invasion, and metastasis^12–16^. Our previous bioinformatics analysis of several public gene expression profiles showed that STX2 upregulation was correlated with a poor prognosis for CRC patients and could increase the activity of the NF-κB signaling pathway^17^. However, the molecular mechanisms through which STX2 regulates NF-κB signaling pathway activation remain unclear.

In this study, we delineate the role of STX2 in CRC metastasis and explore a new molecular mechanism whereby the NF-κB signaling pathway is constitutively activated by STX2. Our findings may provide a potential prognostic biomarker and therapeutic target for combating CRC metastasis.

## Results

### Upregulation of STX2 was associated with the metastasis and poor clinical outcome of CRC

**Fig. 1:**
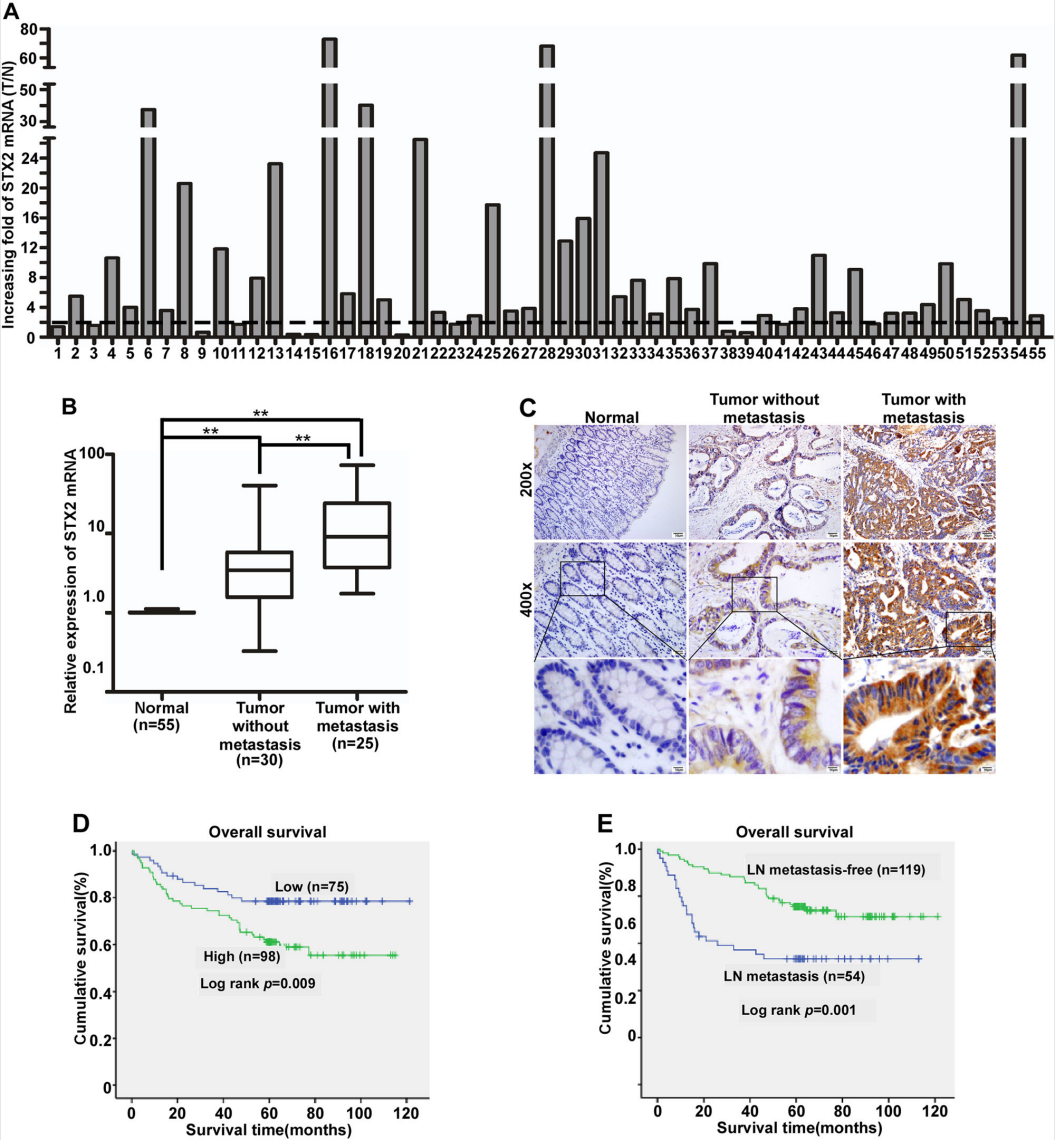
STX2 upregulation was associated with CRC metastasis and a poorer clinical outcome. **a** qPCR analysis of STX2 expression in the fresh human CRC tissues of 55 CRC cases and the matched adjacent normal tissues; STX2 expression was normalized to GAPDH and expressed relative to the matched adjacent normal tissues (2-ΔΔCт). **b** qPCR analysis of STX2 mRNA expression in the normal intestinal mucosa and in primary CRC with or without metastasis. Boundaries of boxes represent bounding of the boxes and stand for the lower and upper quartile. Lines within the boxes and whiskers represent median and extremum(maximum and minimum). **c** IHC analysis of STX2 protein expression in the normal intestinal mucosa and in primary CRC with or without metastasis. **d**, **e** Kaplan–Meier analysis of the influence of STX2 expression and LN metastasis on overall survival. **p < 0.01

We first analyzed STX2 expression in a public database, Oncomine (www.oncomine.com), and found that STX2 was upregulated in CRC compared with matched normal tissues (Figure S1A). The investigation of STX2 expression in GSE41568 and GSE41258 showed that the expression level of STX2 was much higher in CRC with metastasis than in CRC without metastasis (Figure S1B-C). We detected STX2 mRNA expression in 55 primary colorectal tumors and paired adjacent normal tissues using qPCR. A marked (more than twofold) upregulation of STX2 was detected in most of the CRC cases (43/55) (Fig. 1a). Student’s *t*-test showed that STX2 mRNA was upregulated in CRC tissues, especially in the samples with metastasis, compared to the normal tissues (Fig. 1b). The results of IHC revealed that the STX2 protein was mainly localized to the cytomembrane and cytoplasm of the tumor cell (Fig. 1c). Further analysis showed that STX2 protein expression was correlated with the Ducks stage (*p* = 0.000), T classification (*p* = 0.011), N classification (*p* = 0.000) and M classification (*p* = 0.001) of CRC (Table 1 and Table S1). Kaplan–Meier survival analysis indicated that the patients with high-STX2 expression had significantly poorer overall survival (*p* = 0.009) than the patients with low-STX2 expression (Fig. 1d). In addition, the patients with lymph node (LN) metastasis clearly had poorer overall survival (*p* = 0.001) than the patients without LN metastasis (Fig. 1e). Together, these data showed that a close association was evident between STX2 upregulation and CRC metastasis and poorer outcomes for CRC patients.

**Table 1:** Clinicopathologic characteristics of STX2 expression in CRC patients

Characteristics	STX2 expression		*χ*^2^-value	*p*-value
	Low	High		
*Age*				
<60	38	44	0.567	0.539
≥60	37	54		
*Gender*				
Male	42	55	0.063	0.802
Female	33	43		
*Differentiation*				
Well	33	33	6.222	0.045
Moderate	35	42		
Poor	7	23		
*Ducks stage*				
Ducks A	11	3	30.927	0.000
Ducks B	56	49		
Ducks C	8	32		
Ducks D	0	14		
*T classification*				
T1	2	1	11.080	0.011
T2	10	5		
T3	15	8		
T4	48	84		
*N classification*				
N0	65	54	19.715	0.000
N1–2	10	44		
*M classification*				
M0	75	84	11.658	0.001
M1	0	14		

### Overexpression of STX2 promoted the metastasis of CRC cells in vitro and in vivo

**Fig. 2:**
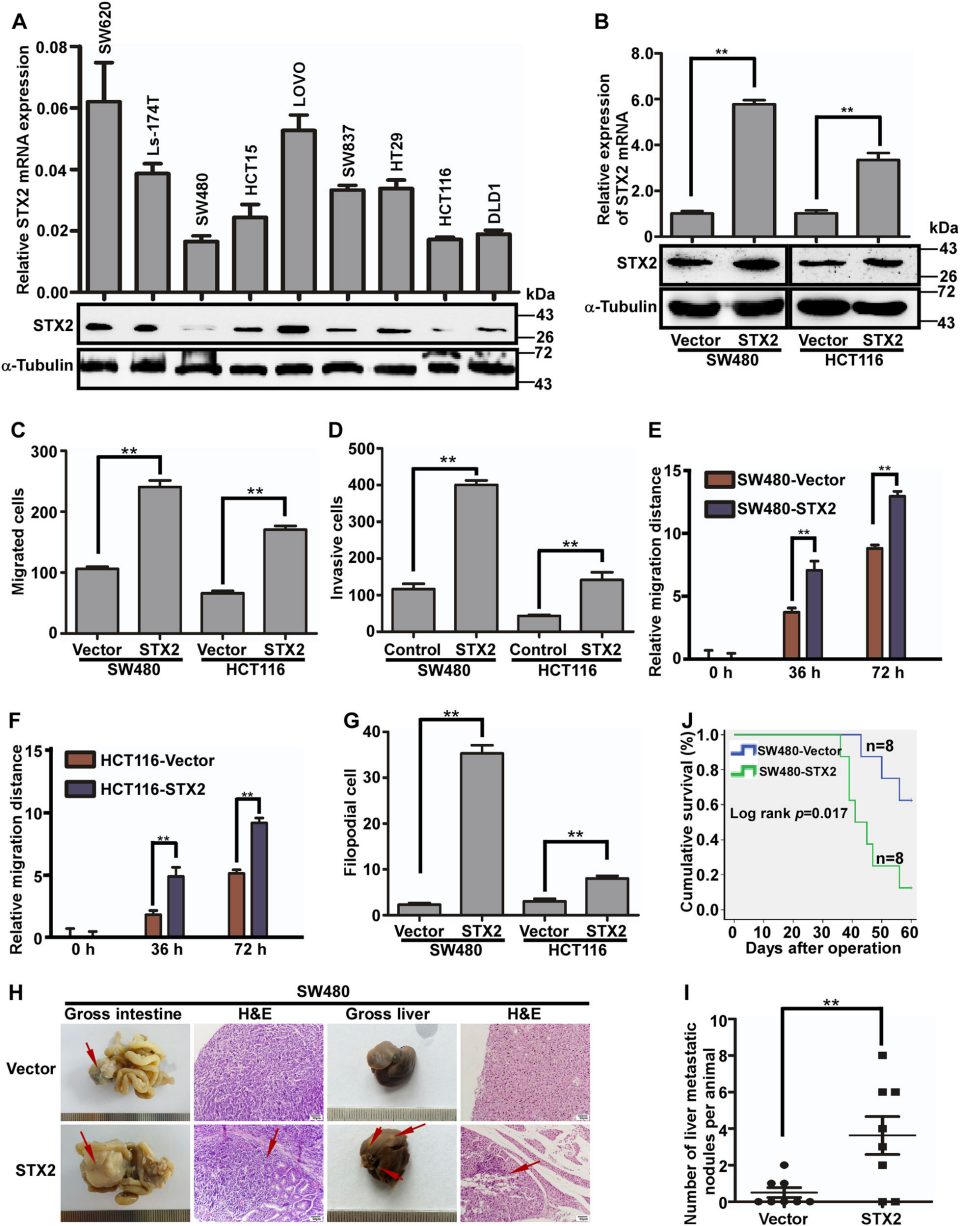
Overexpression of STX2 promoted the metastasis of CRC cells in vitro and in vivo. **a** qPCR and western blot analyses of endogenous STX2 expression in the CRC cell lines. **b** qPCR and western blot analyses of STX2 overexpression in SW480 and HCT116 cells. **c**, **d** Analysis of the migratory and invasive properties of SW480/Vector, SW480/STX2, and HCT116/Vector, HCT116/STX2 cells using Boyden chambers or Matrigel-coated Boyden chambers, respectively. Error bars represent the mean ± s.d. from three independent experiments. **e**, **f** Wound-healing assay. Histograms represent the average migrated distances at the indicated times. Error bars represent the mean ± s.d. from three independent experiments. **g** Three-dimensional morphology analysis. Histograms represent the average number of filopodia formed by each cell sphere from three independent experiments. Error bars represent mean ± s.d. **h** Representative gross and microscopic images of the intestines and livers are shown. The sections were stained with H&E. The arrows indicate the primary tumors in the intestines, visible metastatic nodules in the liver and the infiltration of the primary tumors into the intestines. **i** The number of visible metastatic nodules in the liver. **j** Overall survival time of the mice bearing liver metastases of SW480/Vector and SW480/STX2 tumors. **p < 0.01

The above results suggested that STX2 might play critical roles in CRC metastasis. Therefore, we evaluated the functions of STX2 in CRC cells using both in vitro and in vivo assays. We first investigated the endogenous levels of STX2 in different CRC cell lines and modulated their STX2 levels using STX2 cDNA or lentivirus-mediated STX2-specific short hairpin (sh) RNAs. We found that the STX2 levels in the LOVO and SW620 cell lines, which had higher invasive and metastatic capabilities, increased significantly compared with the cell lines with lower metastatic potentials, such as SW480 and HCT116 (Fig. 2a). We generated exogenous STX2-overexpressing cells by transfection of STX2 cDNA into STX2-low cell lines, namely, SW480 and HCT116 (Fig. 2b). Then, we observed significant increases in the invasion and metastasis of the STX2-overexpressing cells in vitro by Transwell migration, Matrigel invasion, wound-healing and three-dimensional morphogenesis assays (Fig. 2c–g and Figure S2A-E). To further verify the in vivo effect of STX2 on promoting CRC invasion and metastasis, tumors derived from SW480/Vector or SW480/STX2 cells were orthotopically implanted into the ceca of nude mice (*n* = 8 per group). As shown in Figure 2h–i, the SW480/STX2 group had larger primary tumors with obvious invasion to the surrounding tissues in the cecum and much more visible metastatic nodules in the liver than the SW480/Vector group. In addition, the overall survival rate of the liver metastasis-bearing mice was markedly shortened with STX2 overexpression (Fig. 2j).

### Downregulation of STX2 repressed the metastatic potential of CRC cells in vitro and in vivo

**Fig. 3:**
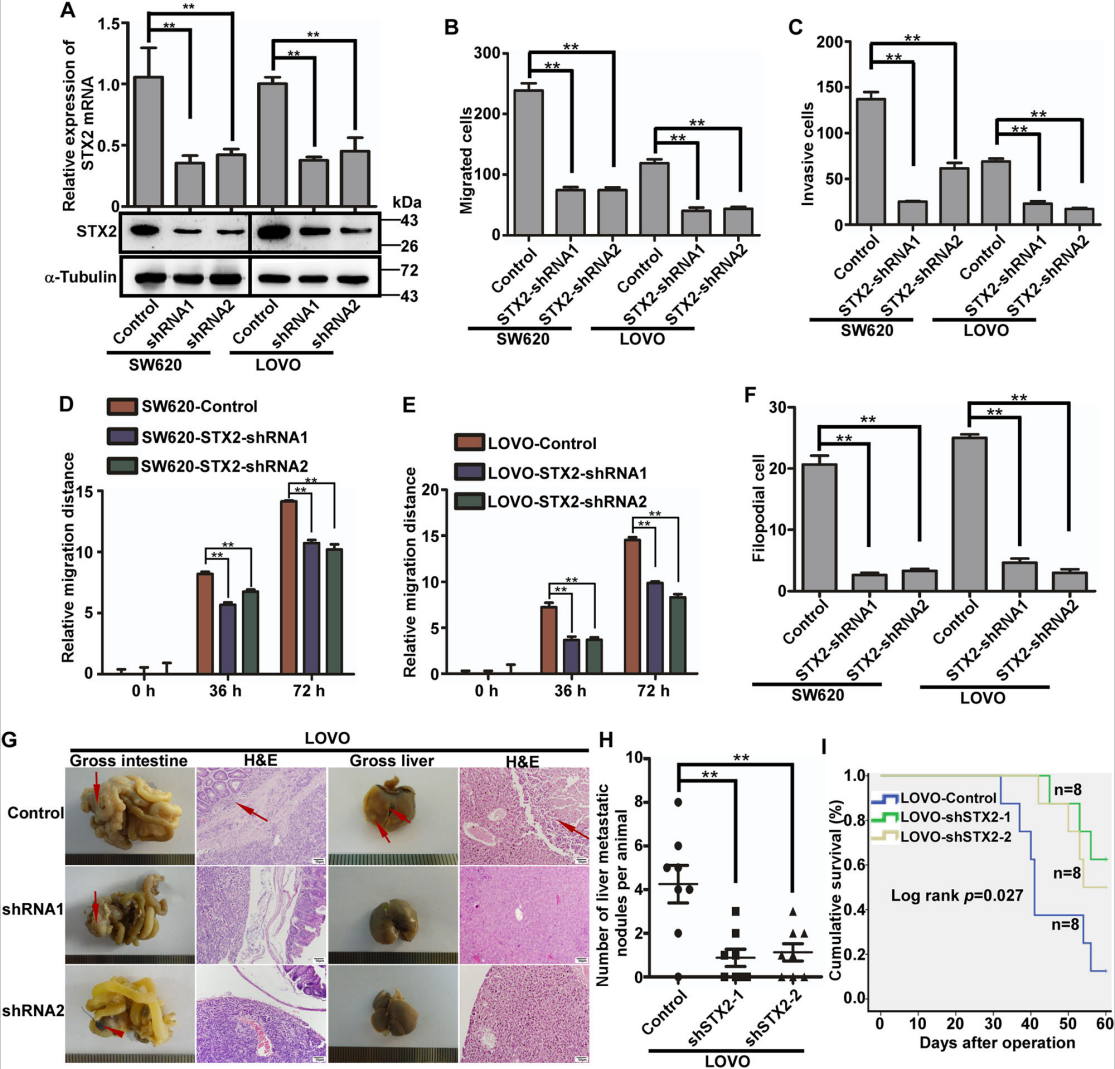
Downregulation of STX2 repressed the metastatic potential of CRC cells in vitro and in vivo. **a** qPCR and western blot analyses of STX2 in specific shRNA-transduced stable cells derived from SW620 and LOVO. **b**, **c** The migratory and invasive properties of the stable cell lines were analyzed using Boyden chambers or Matrigel-coated Boyden chambers. Error bars represent the mean ± s.d. from three independent experiments. **d**, **e** Wound-healing assay. Histograms represent the average migrated distances at the indicated times. Error bars represent the mean ± s.d. from three independent experiments. **f** Three-dimensional morphology analysis. Histograms represent the average number of filopodia formed by each cell sphere from three independent experiments. Error bars represent the mean ± s.d. **g** Representative gross and microscopic images of the intestines and livers are shown. The sections were stained with H&E. The arrows indicate the primary tumors in the intestines, visible metastatic nodules in the liver and infiltrative tumors in the intestine. **h** The number of visible metastatic nodules in the liver. **i** Overall survival times of the different group of mice bearing liver metastases. **p < 0.01

To further validate the role of STX2 in CRC invasion and metastasis, we silenced endogenous STX2 expression in CRC cells using two-specific short hairpin RNAs (shRNAs) (Fig. 3a). The shSTX2-induced inhibition of STX2 resulted in a considerable inhibitory effect on the invasion and metastasis of STX2-high cells in vitro by Transwell migration, Matrigel invasion, wound-healing and three-dimensional morphogenesis assays (Fig. 3b–f and Figure S3A-E). Next, we orthotopically transplanted tumors derived from LOVO cells expressing shRNAs against STX2 or control cells (*n* = 8 per group). The LOVO cells with low-STX2 expression showed smaller primary tumors without obvious invasion to the surrounding tissues in the cecum and less visible metastatic nodules in the liver than the LOVO/Control group (Fig. 3g, h). In addition, the overall survival rate of liver metastasis-bearing mice was significantly extended with the inhibition of STX2 (Fig. 3i).

### STX2 activated the NF-κB signaling pathway

**Fig. 4:**
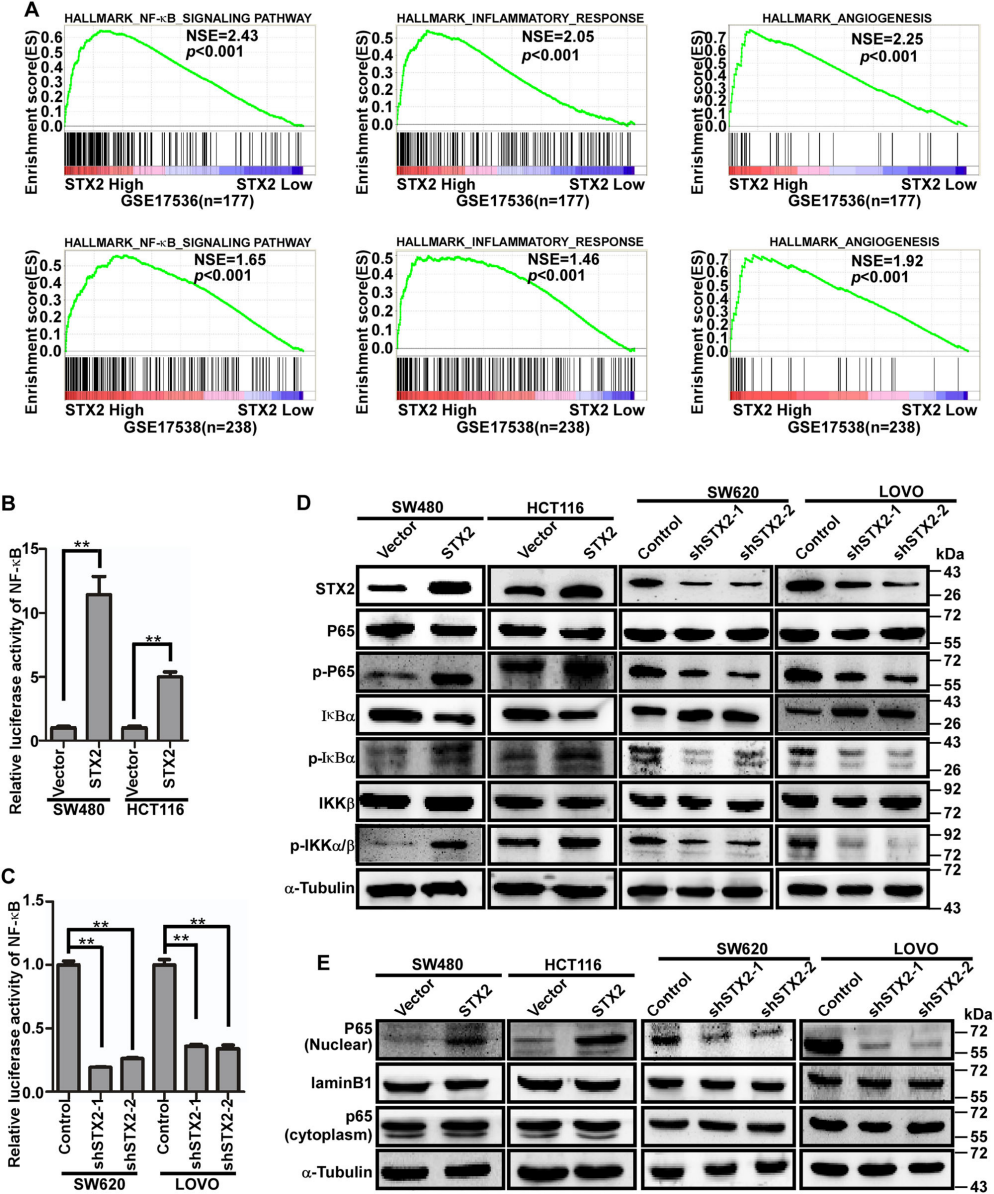
STX2 activated the NF-κB signaling pathway by interacting with TRAF6. **a** Gene set enrichment analysis (GSEA) using GSE13294 and GSE35896. **b**, **c** NF-κB luciferase activity measured in CRC cells with changes in STX2 expression. **d** Western blot analysis of key proteins in the NF-κB pathway in the indicated cells. **e** Western blot analysis of nuclear and cytoplasmic P65 protein expression in the indicated cells. **p < 0.01

To explore the molecular mechanism of STX2-induced CRC invasion and metastasis, gene set enrichment analysis (GSEA)^18^ was conducted. The results of the GSEA assays suggested that multiple malignancy-associated proliferation and metastasis pathway-related genes sets were enriched significantly, including the NF-κB, inflammatory and angiogenesis signaling pathways (Fig. 4a). Activation of the NF-κB signaling pathway is closely correlated with inflammation, tumor growth, and tumor angiogenesis. Therefore, we postulated that STX2 promoted CRC metastasis via the NF-κB signaling pathway. This hypothesis was supported by the following results. The luciferase reporter assays revealed that the luciferase activity of NF-κB was increased in SW480 and HCT116 cells with STX2 overexpression but decreased in SW620 and LOVO cells with STX2 knockdown compared with the paired controls (Fig. 4b, c). In addition, the phosphorylated levels of IKKα/β, p65 (Ser 536) and IκBα increased in the SW480 and HCT116 cells with STX2 overexpression compared with the corresponding controls; however, the expression of phosphorylated IKKα/β, p65, and IκBα decreased in the SW620 and LOVO cells with STX2 knockdown compared with the corresponding controls (Fig. 4d). The results of the western blot analysis of nuclear P65 showed the same trend (Fig. 4e). The above results demonstrated that the activity of the NF-κB signaling pathway could be increased by the upregulation of STX2.

### STX2 activated the NF-κB signaling pathway by interacting with TRAF6

**Fig. 5:**
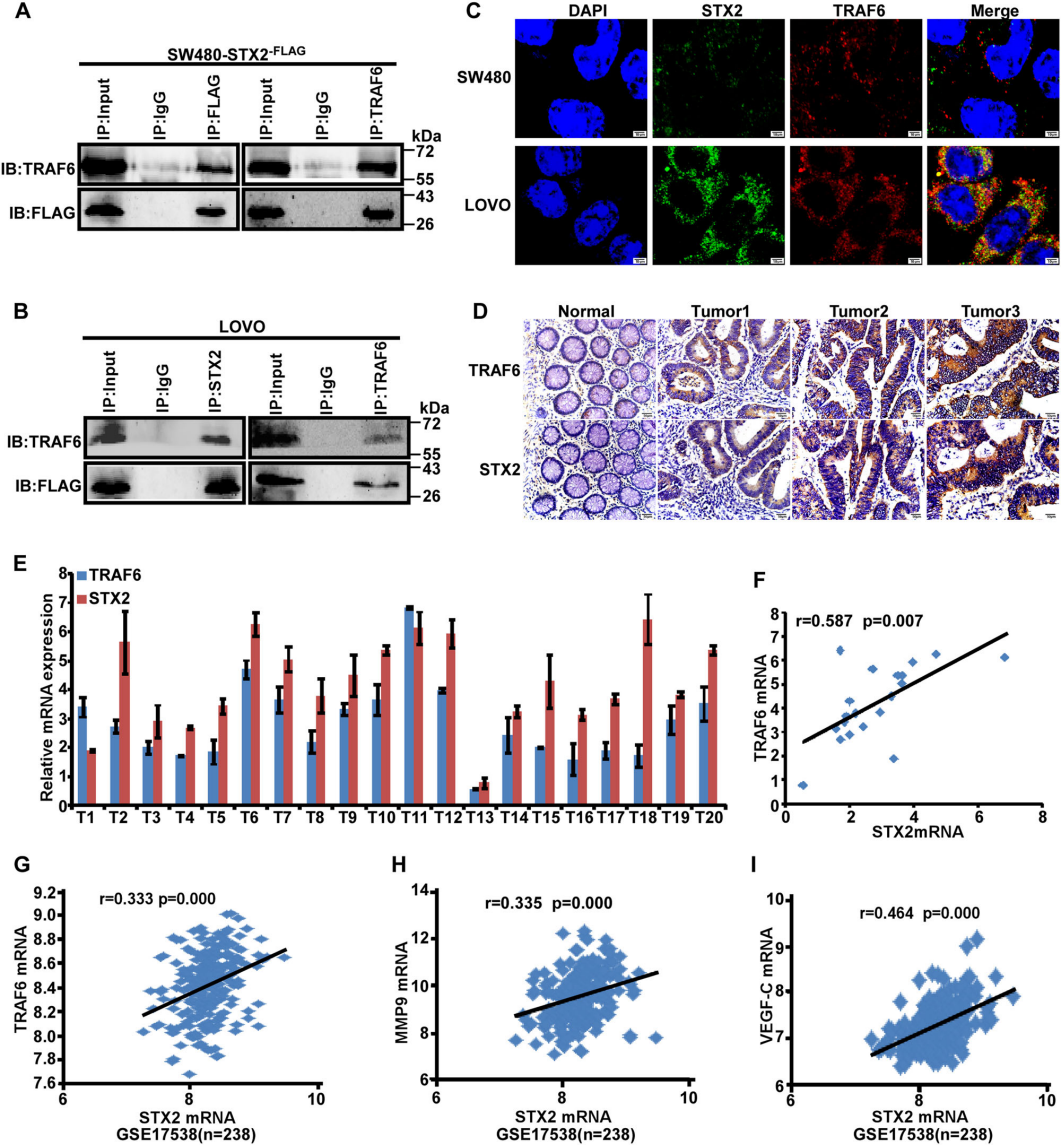
STX2 activated the NF-κB signaling pathway by interacting with TRAF6. **a**, **b** The interaction between STX2 and TRAF6 in CRC cells was determined by Co-IP analysis. **c** The co-localization of STX2 and TRAF6 in CRC cells by IF. **d** IHC analysis of STX2 and TRAF6 protein expression in the same CRC samples. **e** The mRNA expression levels of STX2 and TRAF6 were measured by real-time PCR (2^−ΔCт^). **f** Spearman correlation analysis of STX2 and TRAF6 mRNA expression. **g**–**i** Correlation analysis between STX2 and TRAF6, MMP9, and VEGF-C mRNA expression in GSE17538

To further elucidate the molecular mechanisms of STX2 in activating the NF-κB signaling pathway, Co-IP analysis was conducted and revealed that there was protein–protein interaction between STX2 and TRAF6 both in exogenous and endogenous (Fig. 5a, b). In addition, we also found that there was the same subcellular localization of STX2 and TRAF6 in CRC cells by IF (Fig. 5c). Moreover, IF results also supported a substantial amount of TRAF6 predominantly co-localized with STX2 positive. We analyzed the correlation between the mRNA expression of STX2 and TRAF6 in 20 freshly collected CRC biopsies by qRT-PCR and found that a positive correlation existed between the mRNA expression of STX2 and TRAF6 (Fig. 5e, f). IHC analysis revealed that STX2 expression was positively correlated with TRAF6 protein expression in the CRC tissue samples from 100 cases (Fig. 5d and Table S2). The expression data in GSE13538 also showed that the mRNA expression of STX2 was correlated with the mRNA expression of TRAF6, MMP9, and VEGF-C (Fig. 5e–i). Collectively, these data showed that STX2 activated the NF-κB signaling pathway by interacting with TRAF6.

### NF-κB increased STX2 expression by directly binding to the STX2 promoter

**Fig. 6:**
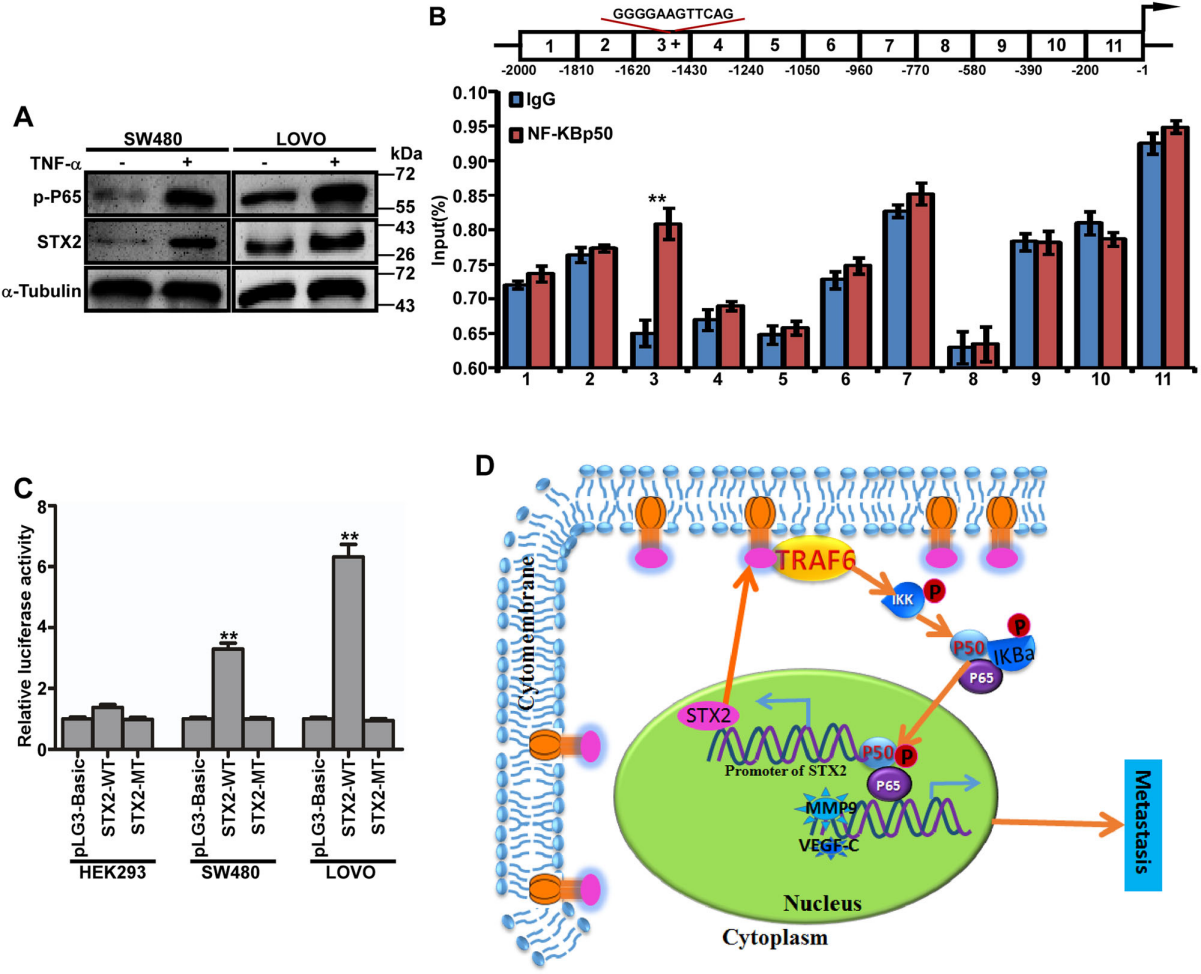
NF-κB increased STX2 expression by directly binding to the STX2 promoter. **a** Western blot analysis of the P65 and STX2 protein levels in SW480 and LOVO cells that had been treated with TNFα (20 ng/ml) for 24 h. **b** ChIP analysis. Schematic illustration of the STX2 promoter. (The region that binds with NF-κB p50 is indicated with “+”; top). ChIP analysis of NF-κB p50 binding with the STX2 promoter in LOVO cells (bottom). **c** Luciferase activity analysis of the indicated cells transfected with the indicated plasmids (Error bars represent the mean ± s.d. from three independent experiments. **d** Model: STX2 increased the activity of the NF-κB pathway by interacting with TRAF6, led to the increased expression of STX2 and formed a positive feedback loop, ultimately promoted CRC metastasis.**p < 0.01

Multiple transcription factor binding sites (TFBS), including sites for NF-κB, were present in the STX2 promoter (Figure S4A). We measured the levels of pP65 and the STX2 protein in CRC cells that had been treated with TNFα and found that both the p-P65 and STX2 protein levels had increased remarkably (Fig. 6a). This result implied that STX2 expression could be upregulated by the activation of NF-κB. Because NF-κB p50 was the main binding subunit of NF-κB, we first performed ChIP assays in LOVO cells to identify the putative binding sites in the STX2 promoter and found that the binding site was located in the −1620 bp~−1430 bp region of the STX2 promoter (Fig. 6b and Figure S4B). In addition, PROMO and QIAGEN indicated the following binding sequence: “5′-GGGGAAGTTCAG-3′”. To verify this binding sequence, we constructed wild type and mutant PLG3-Basic vectors with this sequence and transfected these vectors into cells with different NF-κB activity levels. The results demonstrated that “5′-GGGGAAGTTCAG-3′” was the NF-κB p50 binding site in the STX2 promoter (Fig. 6c). Together, these data demonstrated that STX2 increased the activity of the NF-κB pathway, led to the increased expression of STX2 and formed a positive feedback loop (Fig. 6d).

## Discussion

Metastasis is the primary factor that leads to the poor prognosis of CRC^19–22^. CRC metastasis is a complex process that involves multiple events, including a decrease in cell adhesion, the degradation of the extracellular matrix, the enhancement of the migratory ability of the cell, angiogenesis and changes in the tumor microenvironment, among others^23–27^. Moreover, this process is regulated by multiple functional proteins, including MMPS, VEGF, and TPx^28–31^. These proteins function by altering the activities of several intracellular signaling pathways, such as the Wnt, NF-κB, MAPK, and Notch pathways, and ultimately affect the invasion and metastasis ability of CRC^32–36^. Our study revealed that STX2 increases the metastatic capacity of CRC cells and provided new mechanistic insight into the function of STX2.

There has been some reports about the role of STX2 in cancer^9–11^. However, the findings are some contradictory with each other, which indicates that the function of STX2 in tumorigenesis and cancer development remains poorly understood. Therefore, our data, which have verified the STX2 function in CRC invasion and metastasis and explored the underlying mechanism, will be of great significance. Our data showed that STX2 was upregulated and correlated with the poor prognosis of CRC patients. In addition, STX2 promoted the invasion and metastasis of CRC both in vitro and in vivo. Therefore, our study identified STX2 as a key promoter of CRC metastasis and an independent prognostic predictor for CRC patients.

To elucidate the molecular mechanisms of STX2 in promoting CRC metastasis, GSEA was conducted and found that STX2 was associated with the upregulation of gene sets related to the NF-κB, inflammatory, and angiogenesis signaling pathways. The activation of NF-κB plays critical roles in different processes associated with malignant progression, including tumorigenesis, angiogenesis, invasion, and metastasis. Consequently, we speculated that STX2 might promote CRC metastasis by activating the NF-κB signaling pathway. Our study demonstrated that STX2 increased the luciferase activity of NF-κB, the expression of pP65 and the nuclear translocation of P65. Together, these data confirm that STX2 promotes CRC metastasis by activating the NF-κB signaling pathway.

In addition, our study demonstrated that STX2 protein was co-localized with TRAF6 through protein–protein interaction. TRAF6 is a member of the TRAF family^37,38^. Additionally, TRAF6 is a key molecular regulator of the NF-κB signaling pathway and lies in the upstream of IKK. The results explained the reason that the expression of p-IKK could be regulated by STX2 to some extent. Moreover, TRAF6 plays crucial parts in bone metabolism, homeostasis, and development^39–42^. Recently, studies have increasingly demonstrated that TRAF6 plays vital roles in the tumorigenesis and development of multiple cancers, including CRC^43–47^. In our study, we found that the expression of TRAF6 was positively correlated with the expression of STX2 in CRC tissues. Thus, STX2 promotes CRC metastasis by interacting with TRAF6 and activating the NF-κB signaling pathway.

It has been shown that many TFBS, including NF-κB, were present in the STX2 promoter. To explore the binding sites, ChIP analysis was performed. Our data ultimately revealed that the activation of NF-κB could upregulate the expression of STX2 by directly binding to the STX2 promoter. Consequently, the upregulation of STX2 increased the activity of the NF-κB pathway. In turn, NF-κB increased the expression of STX2, thereby forming a positive signaling loop and leading to the persistent activation of NF-κB in CRC. Based on these, we described an STX2-mediated positive feedback regulatory loop of the NF-κB pathway. This could be a novel finding on the regulatory mechanism of the NF-κB pathway and CRC metastasis. To our knowledge NF-κB pathway is the central player of many biological processes and the persistent activation of NF-κB plays important roles in the tumorigenesis and progression of many tumors^36,48–50^. Therefore, our data provide new mechanistic insight into the critical roles of STX2 toward promoting CRC metastasis.

In summary, our study identified STX2 as a key promoter of CRC metastasis and showed that STX2 promoted CRC metastasis via a positive feedback loop that activating the NF-κB pathway. We propose that the knockdown STX2 will block the persistent activation of NF-κB and ultimately inhibit the metastasis of CRC. And STX2 might be a prognostic marker and therapeutic target for combating CRC metastasis. Of course, to achieve a better understanding of the intrinsic mechanism of STX2-induced CRC metastasis, further molecular studies are necessary for the exploration of the interaction of STX2 and TRAF6 expression in CRC.

## Materials and methods

### Tissue specimens

Fresh CRC biopsies and their matched adjacent normal tissues were collected from 55 cases at the Department of General Surgery, Nanfang Hospital (Guangzhou City, Guangdong Province, China) from February to July 2014. All tissues were freshly frozen in liquid nitrogen until further use. Formalin-fixed paraffin-embedded CRC samples from 173 cases were collected with corresponding clinical medical records at the Department of Pathology, Nanfang Hospital Southern Medical University from January 2000 to December 2005. The patients were followed up by phone regularly to check on their health statuses. None of the patients underwent chemotherapy, radiotherapy, or immunotherapy before surgery. All cases were diagnosed with colorectal columnar adenocarcinoma by Hematoxylin-Eosin (H&E) staining. Prior approval for the use of human tissues and clinical materials was obtained from the Southern Medical University Institutional Board (Guangzhou City, Guangdong Province, China).

### Immunohistochemistry

Paraffin-embedded specimens were cut into 4-μm sections and baked at 60 °C for 2 h. Immunohistochemistry was performed via SP-9000 detection kits that were purchased from ZSGB-BIO (Beijing, China). The slides were incubated overnight at 4 °C with a primary antibody, namely, rabbit polyclonal anti-STX2 (1:200, Proteintech, USA), or mouse polyclonal anti-tumor necrosis factor receptor-associated factor (TRAF6) (1:200, Sigma, USA). As the negative control, PBS was used in place of the primary antibody. The sections were incubated with 3,3-diaminobenzidine (DAB) for 1 min and counterstained with Hematoxylin and dehydrated with a gradient alcohol series and xylenes. The slides were sealed with neutral balsam. The sections were evaluated and scored independently by two researchers, who were blind to the patient outcomes, based on both the proportion of positively stained tumor cells and the intensity of the staining^51^.

### Cell culture

The human CRC cell lines such as SW620, Ls-174T, SW480, HCT15, LOVO, SW837, HT29, HCT116, and DLD1 were obtained from the American Type Culture Collection (ATCC, USA). SW620 and SW837 were cultured in DMEM (Invitrogen, USA) with 10% fetal bovine serum (FBS, Gibco), and Ls-174T, SW480, HCT15, LOVO, HT29, HCT116, and DLD1 were maintained in RPMI-1640 medium (Invitrogen, USA) with 10% FBS at 37 °C in a humidified atmosphere with 5% CO_2_.

### RNA extraction and real-time quantitative PCR (qPCR)

Total RNA from human tissues and cultured cells was extracted with the TRIzol reagent (Invitrogen, USA) according to the manufacturer’s instructions. Reverse transcription (RT) was carried out according to the manufacturer’s protocol. qPCR was conducted with SYBR Green I (Applied BioSystems, USA). The data were normalized to the geometric mean of the housekeeping gene, namely, GAPDH, and calculated as 2^–ΔΔCT^. The primer sequences are shown in Supplementary Table 3 (Table S3).

### Western blot and co-immunoprecipitation (Co-IP)

Protein lysates were subjected to SDS-PAGE, and the resolved proteins were then transferred to PVDF membranes. The membranes were blocked in 5% non-fat dry milk or bovine serum albumin/fraction V (BSA, Sigma, USA) and then incubated overnight at 4 °C with the following primary antibodies: STX2 (Proteintech, USA), TRAF6 (Proteintech, USA), IKKβ (CST, USA), pIKKα/β (CST, USA), IκBα (CST, USA), pIκBα (CST, USA), P65 (CST, USA), pP65 (CST, USA) and α-Tubulin (Sigma, USA). Finally, the appropriate secondary antibodies (HRP-conjugated anti-rabbit IgG (CST, USA) and HRP-conjugated anti-mouse IgG (CST, USA) were applied. Chemiluminescent signals were detected with SuperSignal West Pico and exposed to autoradiography (HyBlot CL). α-Tubulin was used as the internal control to confirm the equal loading of the proteins.

Cell lysates for Co-IP were prepared from SW480-STX2^-Flag^ and LOVO. The cell lysates were pre-cleared by incubating with protein A + G Sepharose beads (Sigma, USA). Then, the individual antibodies for STX2, TRAF6, and IgG were added to the lysates, and the samples were incubated overnight at 4 °C, after which the complexes were collected with protein A + G Sepharose beads and brief centrifugation. Finally, the bound proteins were separated by SDS-PAGE.

### Immunofluorescence analysis

The cells were seeded on coverslips at a density of 5 × 10^4^ per well for 48 h and then were incubated with a primary antibody STX2 (1:100, Proteintech, USA) or TRAF6 (1:100, Sigma, USA). The coverslips were then incubated with rhodamine-conjugated or fluoresceinisothiocyanate (FITC)-conjugated goat antibodies against rabbit or mouse IgG (anti-rabbit IgG, Abcam; anti-mouse IgG, Abcam). After counterstaining with 4,6-diamidino-2-phenylindole (DAPI; Sigma), images were taken using an Olympus FV1000 confocal laser-scanning microscope.

### Plasmid construction and transfection

Overexpression plasmids for STX2 and STX2^-Flag^ were generated by cloning PCR-amplified full-length human STX2 cDNA into pSin-EF-2 (Addgene, USA). The primers used to construct the plasmids are listed in Supplementary Table 4 (Table S24). Human STX2 siRNAs were purchased from Ribobio (Guangzhou, China). After screening in CRC cells after transient transfection, we identified the effective interfering sequences and constructed a recombinant lentiviral vector with GV248 (GeneChem, China) (Table S5). Lentivirus was produced by HEK293FT cells using the calcium phosphate method. Transfected cells were selected in medium containing puromycin. mRNA and protein were extracted from the samples for the qPCR and western blot analyses, respectively.

### Transwell migration, Matrigel invasion, wound-healing and three-dimensional morphogenesis assays

Transwell migration, Matrigel invasion, wound-healing and three-dimensional morphogenesis assays were performed with CRC cells transfected with control lentivirus, STX2, or STX2-shRNAs. The details are provided in Supplementary materials and Methods section.

### Luciferase assays

The cells were seeded in 24-well plates (1 × 10^5^/well) the day before transfection. The luciferase reporter plasmid, Renilla luciferase reporter plasmid pRL-TK (Promega, USA), and indicated plasmids were then co-transfected into the cells using the Lipofectamine 2000 reagent (Invitrogen, USA). Forty-eight hours after the transfection, the luciferase and Renilla activities were assessed with the Dual Luciferase Reporter Assay kit (Promega, USA) following the manufacturer’s instructions. The results were presented after normalization with the measured values of firefly luciferase. All experiments were conducted at least three times, and the data are presented as the mean ± standard deviation (mean ± s.d.).

### ChIP assays

ChIP assays were carried out using a kit according to the manufacturer’s instructions (Millipore Corporation, USA). To cross-link chromatin-associated proteins to DNA, ~1 × 10^7^ cells per 100-mm culture dish were treated with 1% formaldehyde for 10 min, and then, glycine was added to quench the formaldehyde. Cells were collected in 2 ml of cell scraping solution with 10 μl of 100 mM phenylmethanesulfonyl fluorides (PMSF). The supernatant was discarded after centrifugation at 2500 r.p.m. for 10 min at 4 °C. One milliliter of lysis buffer with 5 μl of PIC and 5 μl of PMSF was added to break down the large molecules. After centrifugation at 5000 r.p.m. for 10 min at 4 °C, the supernatant was discarded, and 350 μl of shearing buffer with 1.75 μl of PIC and 1.75 μl of PMSF was added. Then, the lysates were sonicated with 40% power for 10-s pulses to shear the DNA to 200–1000-bp fragments. The lysates were cleared by centrifugation at 15,000 r.p.m. for 10 min at 4 °C. For immunoprecipitation, the pre-cleared lysates containing 25 μg of DNA mixed with ChIP buffer I, PIC and 25 μl of Protein G magnetic beads were incubated with the NF-κB P50 antibody or the normal mouse immunoglobulin G as the negative control overnight at 4 °C with rotation. The human STX2 promoter was amplified by qPCR. ChIP assays were performed three times, and the sequences of the qPCR primers are listed in Supplementary Table 6 (Table S6). The sequences of the wild type and mutant vector targets are shown in Supplementary Table 7 (Table S7).

### Animal studies

All animal experiments were conducted in accordance with standard procedures and approved by the institutional Use Committee for Animal Care. A total of 1 × 10^6^ cells were diluted in 200 μl of RPMI-1640 medium without FBS and were then inoculated subcutaneously into the dorsal surface of 4–6-week-old BALB/C nude male mice. The mice were purchased from the Animal Center of Southern Medical University, Guangzhou, China. Two weeks later, the xenografts were excised and minced into 1-mm^3^ pieces. Nude mice were anesthetized and underwent surgical orthotopic implantation using these pieces as previously reported^52^. All mice were housed in specific pathogen-free conditions. The mice were closely observed when significant cachexia was apparent. Then, the survival time was recorded. All mice were killed 60 days after surgery. The individual organs were excised, and the numbers of gross metastatic foci were determined. The organs were fixed in 4% paraformaldehyde and embedded in paraffin, and 4-µm sections were prepared and stained with H&E.

### Statistical analysis

All statistical analyses were performed using SPSS20.0 for Windows. Mann–Whitney *U*-tests were used to analyze the relationship between STX2 expression and the clinicopathologic features of CRC. The non-parametric Spearman method was used to evaluate the correlation between STX2 and TRAF6 expression. Student’s *t*-tests were used to compare the qPCR values between subgroups. Survival curves were determined with the Kaplan–Meier method and compared with the log-rank test. *p* < 0.05 was considered significant. Statistically significant data were indicated by asterisks: *p < 0.05, **p < 0.01.

## Electronic supplementary material

Supplementary Materials

Figure S1

Figure S2

Figure S3

Figure S4
