# Supplementary material for: STX2 promotes colorectal cancer metastasis through a positive feedback loop that activates the NF-κB pathway
Source: Cell Death Dis. 2018 May 31;9(6):664. doi: 10.1038/s41419-018-0675-x (PMC5981218; doi:10.1038/s41419-018-0675-x)
Supplement: Supplementary file 1 — Supplementary Materials [file 41419_2018_675_MOESM1_ESM.doc]

**STX2 promotes colorectal cancer metastasis through a positive feedback loop that activates the NF-κB pathway**

**Supplementary Materials and Methods**

**Transwell migration assay**

For Transwell migration assays, Boyden chambers with filter membranes containing 8-μm pores were used. Briefly, cells (1×10^5^) in culture medium without FBS were seeded to the upper chamber, and culture medium containing 20% FBS as a chemoattractant was added to the lower chamber. After incubation for 48 h, the chamber was fixed in 4% paraformaldehyde and stained with Hematoxylin. Cells on the upper sides of the filters were removed with cotton swabs. Cells that migrated to the lower sides were stained with Hematoxylin. The migratory cells on the lower surfaces of the filters were counted. Three independent experiments were performed, and the data were presented as the mean±s.d.

**Matrigel invasion assay**

Matrigel invasion assays were performed as described above for the Transwell migration assays, except that the upper sides of the filters were pre-coated with 0.2% Matrigel (BD Biosciences, USA). The experiment was repeated three times.

**Wound-healing assay**

Cells were seeded to six-well plates and incubated under permissive conditions until the cells reached 90% confluence. After serum starvation for 24 h, wounds were generated in the confluent cells using a pipette tip. Wound healing within the scrape line was then observed and photographed at indicated time points. Each experiment was repeated at least three times.

**Three-dimensional morphogenesis assay**

Cells (1×10^4^) were resuspended in medium supplemented with 5% Matrigel (BD Biosciences, USA). Then, the Matrigel-cell mixtures were seeded to a 24-well plates pre-coated with Matrigel. The plates were incubated at 37°C in a humidified atmosphere containing 5% CO_2_. Three-dimensional morphological structures were observed, and the microscopic images were captured at 2-day intervals for 2-3 weeks. The filopodia formed by each cell sphere were counted under a microscope.

**Supplementary Tables**

Supplementary Table 1. Spearman correlation between STX2 expression and Clinicopathologic Characteristics

| **Variables** | **STX2 Expression** | |
| --- | --- | --- |
|  | **Spearman correlation** | **p value** |
| **Age** | 0.057 | 0.454 |
| **Gender** | 0.024 | 0.756 |
| **Differentiation** | 0.161 | 0.035 |
| **Ducks stage** | 0.421 | 0.000 |
| **T classification** | 0.252 | 0.001 |
| **N classification** | 0.387 | 0.000 |
| **M classification** | 0.260 | 0.001 |

Supplementary Table 2. Correlation between STX2 and TRAF6 expression in CRC tissues by IHC

| **STX2** | **TRAF6** | | **r** | **p** |
| --- | --- | --- | --- | --- |
|  | **High expression** | **Low expression** |  |  |
| **High expression** | 50 | 8 | 0.629 | 0.000 |
| **Low expression** | 10 | 32 |  |  |

Supplementary Table 3. Primer sequences used for qRT-PCR

| **Gene** | **Forward primer** | **Reverse primer** |
| --- | --- | --- |
| **STX2** | TGAGAGTGGGAACCGGACTT | TTCTAGCTCGTCGTCTGTGGT |
| **TRAF6** | AACTGTGCTGCATCAATGGC | CCCAGAGTCGGGTATAACGC |
| **GAPDH** | GACTCATGACCACAGTCCATGC | AGAGGCAGGGATGATGTTCTG |

Supplementary Table 4. Primer sequences used for plasmid construction PCR

| **Gene** | **Forward primer** | **Reverse primer** |
| --- | --- | --- |
| **STX2** | ATGCGGGACCGGCTGCCAGA | TCATTTGCCAACTGACAAGC |
| **STX2-Flag** | CGCGGATCCATGCGGGACCGGCTGCCAGA | CCGGAATTCTCATTTGCCAACTGACAAGC |

Supplementary Table 5. The sequences specifically targeting STX2

| **Gene** | **sequences specifically targeting STX2 and TRAF6** |
| --- | --- |
| **STX2-shRNA1** | CCACACGTTTGCAACCCAA |
| **STX2-shRNA2** | CCTCCTGGTATCTGAACCA |

Supplementary Table 6. Primer sequences used for ChIP qPCR

| **STX2-CHIP-primer** | **Sequence (5’to 3’)** |
| --- | --- |
| **STX2 -1-F** | **GAACTAATAAATGTCCATGA** |
| **STX2 -1-R** | **CACCTCAGCCTCCAAAAGCA** |
| **STX2 -2-F** | **TGCTTTTGGAGGCTGAGGTG** |
| **STX2 -2-R** | **ACATATGATAATATTGGCAG** |
| **STX2 -3-F** | **CTGCCAATATTATCATATGT** |
| **STX2 -3-R** | **AGATATGTTTTTTCTCTCCA** |
| **STX2 -4-F** | **TGGAGAGAAAAAACATATCT** |
| **STX2 -4-R** | **TCTTGACAGCCAGTTGTCCA** |
| **STX2 -5-F** | **TGGACAACTGGCTGTCAAGA** |
| **STX2 -5-R**  **STX2 -6-F**  **STX2 -6-R** | **AAGGCCAGATTTGTTTTTAA**  **TTAAAAACAAATCTGGCCTT ACTGGGCTCCATTGCATGTG** |
| **STX2 -7-F** | **CACATGCAATGGAGCCCAGT** |
| **STX2 -7-R**  **STX2 -8-F**  **STX2 -8-R** | **AGCCAAGGAAGAAGGGCAGA**  **TCTGCCCTTCTTCCTTGGCT ACAGTCCCCAAGACCATTAT** |
| **STX2 -9-F** | **ATAATGGTCTTGGGGACTGT** |
| **STX2 -9-R**  **STX2 -10-F**  **STX2 -10R** | **GAAGAGCGTCCGCAGGCCCA TGGGCCTGCGGACGCTCTTC**  **TCAACGAATAGTGACAAAAG** |
| **STX2 -11-F** | **CTTTTGTCACTATTCGTTGA** |
| **STX2 -11-R** | **CGGCCCGCGCCGCGCCCCGC** |

Supplementary Table 7. The sequences of wild and mutant vector targets

| **STX2-promoter** | **Sequence (5’to 3’)** |
| --- | --- |
| **STX2-promoter-WT** | GGGGAAGTTCAG |
| **STX2-promoter-MUT** | GGTTCCTGGCAG |

**Supplementary Figure Legends**

**Supplementary Figure 1.** STX2 up-regulation was associated with CRC metastasis and a poor clinical outcome. (A) STX2 expression in CRC was assessed with a public database, Oncomine. (B) STX2 expression in primary CRC with or without metastasis, based on GSE41657. (C) STX2 expression in primary CRC with or without metastasis, based on GSE39582.

**Supplementary Figure 2.** Overexpression of STX2 promoted the metastasis of CRC cells in vitro. (A,B) Representative images of the Transwell migration and Matrigel invasion assays. Original magnification, ×200. (C,D) Representative images of the wound-healing assays. Original magnification, ×100. (E) Representative images of the three-dimensional morphology analysis. Original magnification, ×400.

**Supplementary Figure 3.** Down-regulation of STX2 repressed the metastatic potential of CRC cells in vitro. (A,B) Representative images of Transwell migration and the Matrigel invasion assays. Original magnification, ×200. (C,D) Representative images of the wound-healing assays. Original magnification, ×100. (E) Representative images of the three-dimensional morphology analysis. Original magnification, ×400.

**Supplementary Figure 4.** NF-κB increased STX2 expression by directly binding to its promoter. (A) TFBS in the STX2 gene promoter, as shown in QIAGEN. (B) ChIP analysis of NF-κB p50 binding to the STX2 promoter in CRC cells. The GAPDH promoter was used as the negative control.
