# Supplementary figures and images for: STX2 promotes colorectal cancer metastasis through a positive feedback loop that activates the NF-κB pathway
Source: Cell Death Dis. 2018 May 31;9(6):664. doi: 10.1038/s41419-018-0675-x (PMC5981218; doi:10.1038/s41419-018-0675-x)

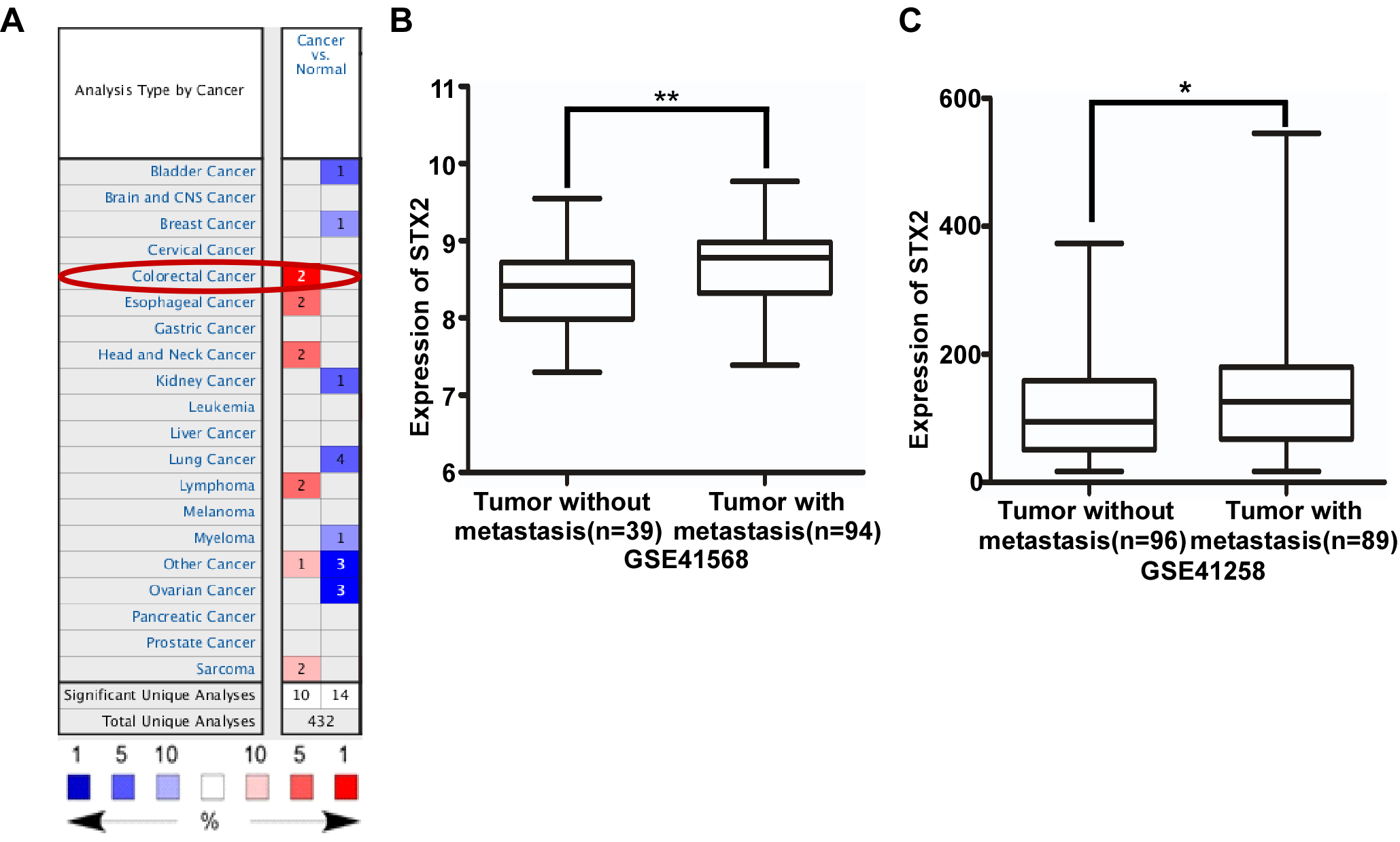

Supplement: Supplementary file 2 — Figure S1 [file 41419_2018_675_MOESM2_ESM.tif]

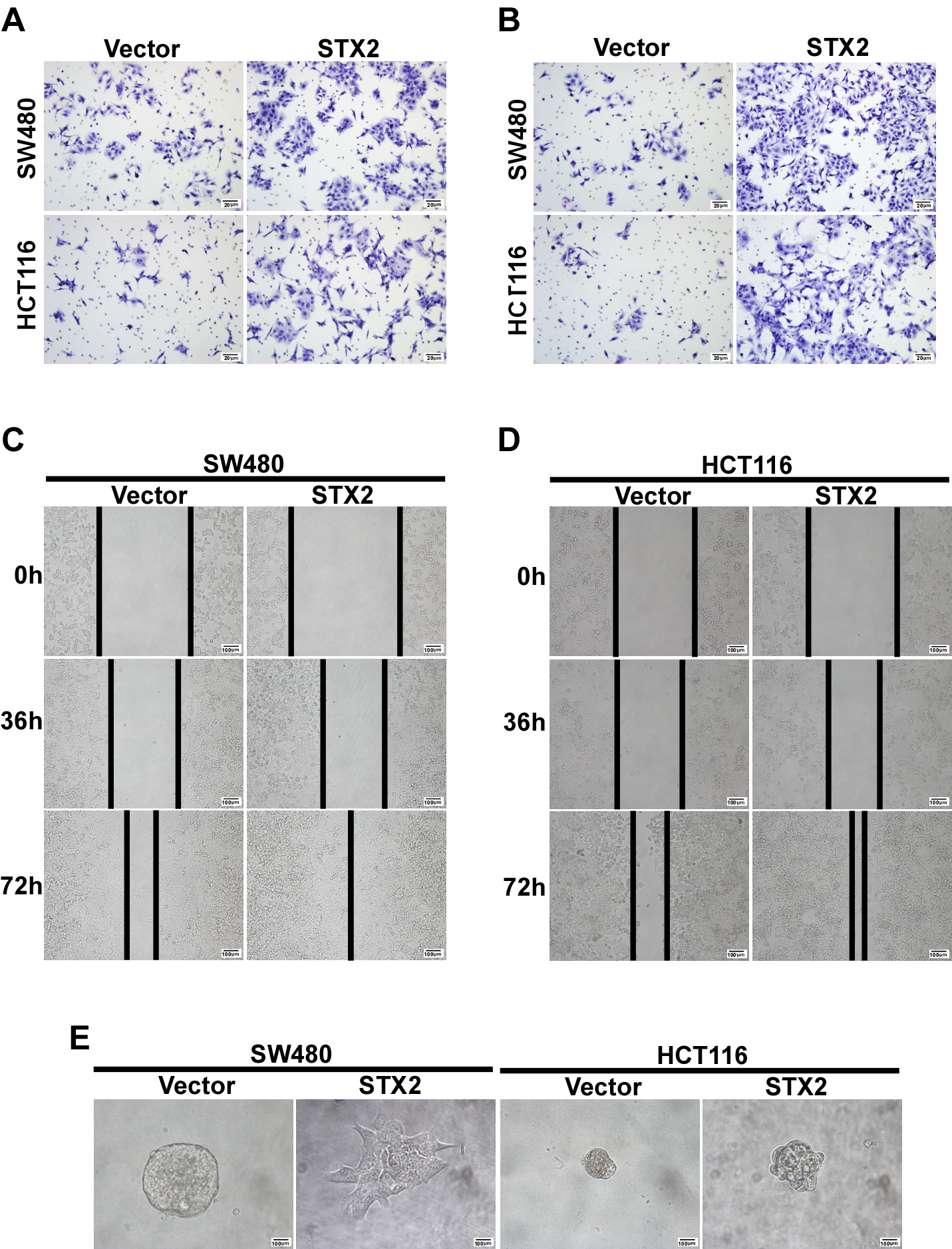

Supplement: Supplementary file 3 — Figure S2 [file 41419_2018_675_MOESM3_ESM.tif]

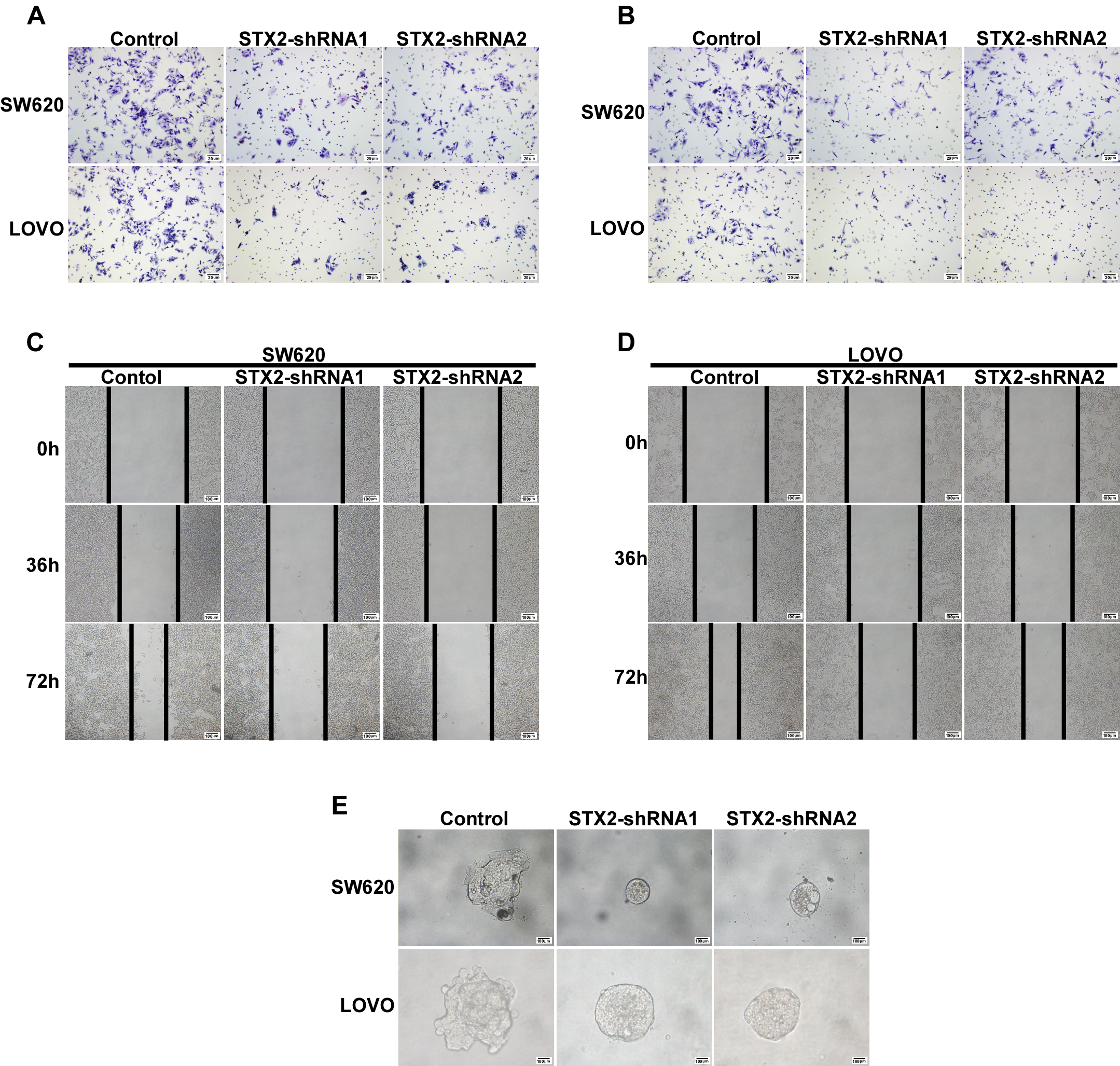

Supplement: Supplementary file 4 — Figure S3 [file 41419_2018_675_MOESM4_ESM.tif]

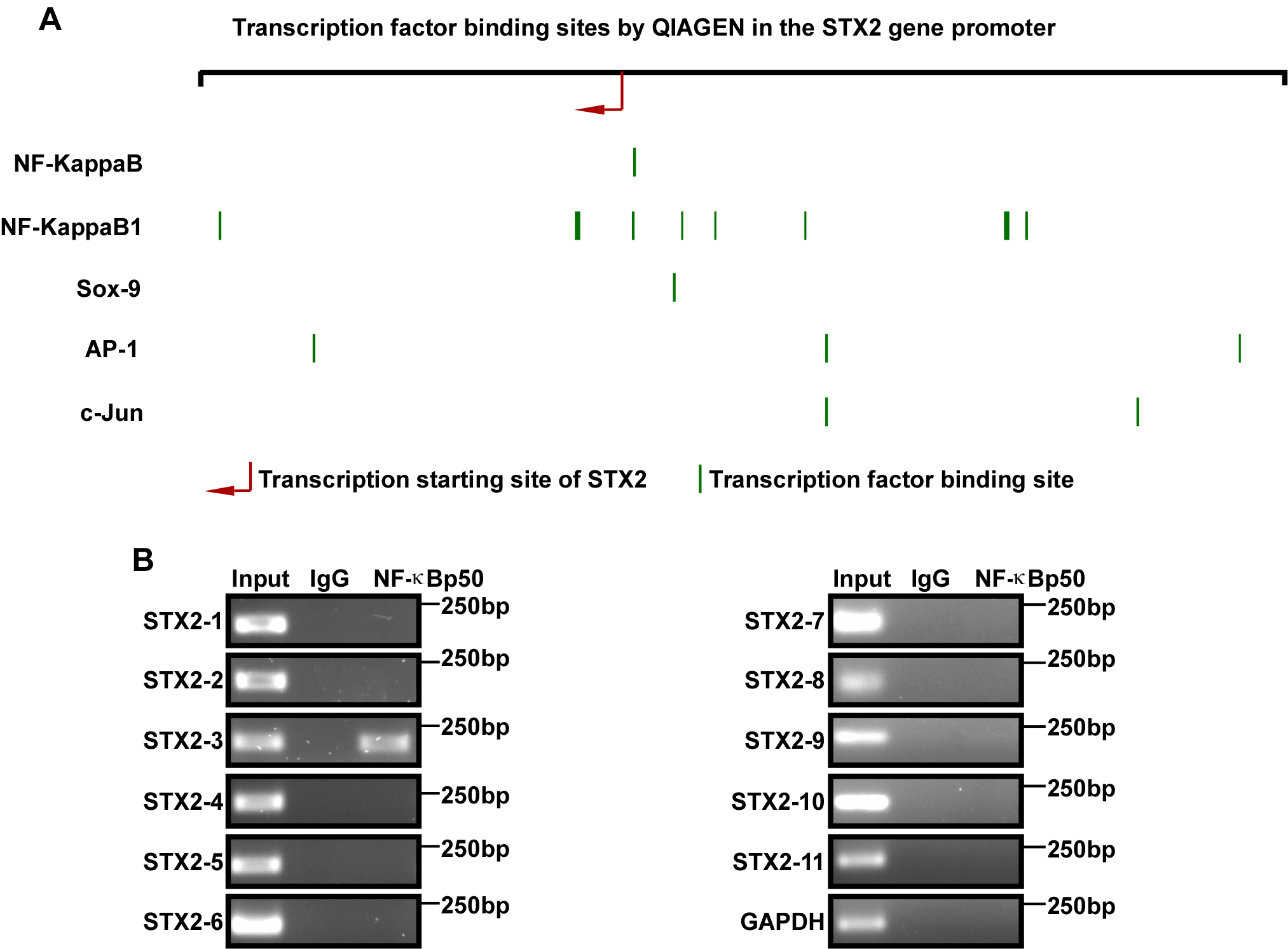

Supplement: Supplementary file 5 — Figure S4 [file 41419_2018_675_MOESM5_ESM.tif]
